# Let’s not steal equity from our patients in the name of quality

**DOI:** 10.3389/fpubh.2025.1522743

**Published:** 2025-06-06

**Authors:** Chelsea D. Hicks, Heather Barnett, Jennifer Shi, Julia Velonjara, Zaher Kmail, Monica S. Vavilala, Edwin G. Lindo

**Affiliations:** ^1^Department of Pediatrics, University of Washington, Seattle, WA, United States; ^2^Harborview Injury Prevention & Research Center, University of Washington, Seattle, WA, United States; ^3^Department of Rehabilitation Medicine, University of Washington, Seattle, WA, United States; ^4^Department of Anesthesiology and Pain Medicine, University of Washington, Seattle, WA, United States; ^5^Department of Statistics, University of Washington-Tacoma, Tacoma, WA, United States; ^6^Department of Family Medicine, Office of Healthcare Equity, University of Washington, Seattle, WA, United States

**Keywords:** health equity, injury, trauma, neurotrauma, healthcare quality

## Abstract

Healthcare can be, we believe, the vanguard to lead us to a more just society but has much work to do. Quality improvement (QI) processes drive care delivery based on metrics and reporting requirements, but equity is not a commonly used QI measure, and the extent of inequitable care affecting patients is unclear at best. While quality metrics can provide benchmarks for healthcare based on published evidence, quality metric standards that do not consider healthcare equity will not lead to the provision of equitable, patient-centered care. In fact, equity is separated from quality in most existing quality metric frameworks when, instead, achieving equity should be a central component of high-quality care. This is true even for leading health conditions, such as injury and violence. Yet, achieving equitable care is every patient’s right and achieving healthcare equity should be a societal and bedside goal. We call for alignment between patients, healthcare providers, and healthcare organizations to unite health equity and healthcare quality metrics. Finally, we offer some recommendations and an example of success in pursuing and operationalizing health equity.

## Introduction

Healthcare can be, we believe, the vanguard to lead us to a more just society, but we have much work to do to make this possibility a reality. In 1966, at the annual meeting for the Medical Committee for Human Rights, Dr. King declared unequivocally, “Of all the forms of inequality, injustice in health is the most shocking and the most inhuman because it often results in physical death” (1). In this perspective, we focus on the need to ensure health equity and build trust between patients, clinicians, and the healthcare system at large.

From a public health perspective, some of the most dramatic health disparities, with some of the greatest need and opportunity for improvement, occur in the realm of trauma and injury care. Unintentional injury is the third leading cause of death in the United States and the leading cause of death among individuals ages 1–44 (2, 3). Disparities between privileged and marginalized populations occur at all levels of trauma, including incidence, severity, healthcare access and interventions, and outcomes (4). Improving equity in trauma care requires proactive efforts throughout the healthcare system. In this perspective, we argue that the current framework of quality measurement and improvement is insufficient to address equity. To address inequities across the healthcare system, redefining the concept of quality and the relationship between quality and equity is essential. A purpose of our viewpoint was to propel development of alternate approaches to use of the Agency for Healthcare Research and Quality (AHRQ) framework of six domains of healthcare quality, one of which is, separately, equity (5).

Defining equity in healthcare quality is not standardized and measures of healthcare equity are not included in commonly used quality improvement measurement tools (6). The AHRQ defines equity as “providing care that does not vary in quality because of personal characteristics such as geographic location and socioeconomic status” and equity is listed as a separate domain than other quality measures. More measurement tools are needed to understand how to consider equity as part of quality of healthcare. Empathy and responsiveness, as well as other mindset attributes, are critical to uphold equity but standing alone, are insufficient without other actions to achieve healthcare equity.

For example, equity may have component attributes that are not captured in the AHRQ domain. One example is shown in the service quality (SERVQUAL) and service performance (SERVPERF) models (7, 8), which demonstrate equity as part of quality by considering tangibles, reliability, responsiveness, assurance, and empathy. The component factors of these models are not currently part of most quality metrics; however, using these metrics to perform factor analysis could identify the features of equity in other quality domains. Considering SERVQUAL and or SERVPERF model components in exploratory and confirmatory factor analytical approaches may help remove the separation of equity from traditionally used quality definitions.

## Relationship between equity and quality measures of healthcare

Trauma care improvement processes drive care delivery based on metrics and reporting requirements, but equity is not a trauma care measure, and because of limited research the extent of inequitable care affecting trauma patients has proven to be unclear at best. While quality metrics can provide benchmarks for trauma care based on published evidence, quality metric standards that do not consider healthcare equity will not lead to the provision of equitable, patient-centered care. In fact, equity is separated from quality in most existing frameworks when, instead, equity should be a central component of high-quality care (9–13).

Trauma care should center equity at each phase and across the continuum of healthcare (9–13). The Institute of Medicine’s (IOM, now National Academy of Medicine) 2000 report “To Err Is Human: Building a Safer Health System” launched the health care quality movement in the US (14). In 2001, “Crossing the Quality Chasm” by the IOM Committee on Quality of Health Care in America outlined strategies for improving quality through key aims of a high-quality healthcare system: safety, effectiveness, patient-centeredness, timeliness, efficiency, and equity (15). This report called to action both health systems and providers to change health care delivery to improve the quality of American healthcare. In 2015, the IOM expanded the initial discussion to address improving diagnosis and reducing diagnostic errors in healthcare (16). Discussions on delivering quality medicines to displaced persons, racial bias in health algorithms (17), as well as the case against race-based glomerular filtration rates have been brought to the forefront to address the need to reconsider how equity is positioned in the quality argument (18). We must do the same across all domains of healthcare, particularly trauma care.

The inclusion of equity as an isolated and separate quality measure is not sufficient. First, inequities are found in each of the other quality measures (safety, effectiveness, patient-centeredness, timeliness, and efficiency). Listing equity as a separate quality measure implies that these other quality measures are devoid of the equity imperative. Second, there is no explicit measurement of equity within these priority measures. While the intention may be to imply that equitable care will be ensured through quality and standardization of care alone, this framing falls short of addressing the ongoing disparities in outcomes as measured by these quality considerations.

We argue that health care quality cannot exist without equity. Equity is an intrinsic part of, and requirement for, quality; therefore, initiatives that report improvement in “quality” while not addressing equity are not only unsuccessful but counterproductive to quality improvement and may ultimately worsen health inequities. Unfortunately, separating quality from equity in healthcare has allowed many quality initiatives to omit equity as a consideration. Traditional quality improvement work has often been performed with the assumption that any benefits will apply broadly across all groups. This assumption has been disproven in multiple studies. When qualitative improvement initiatives are undertaken without an explicit focus on equity, pre-existing disparities may actually worsen (19). Many qualitative improvement initiatives preferentially benefit more privileged populations. Privileged groups may benefit uniquely if they have better access to the intervention, are more likely to adopt and adhere to the intervention, or if the intervention is tailored for them. In public health, this process is known as “intervention-generated inequality” and is well documented (20).

## Responsibility, authority and accountability for delivery of equitable healthcare

Healthcare systems, providers, and staff are expected to engage in regular quality improvement; but without explicit focus on equity and an understanding of the intrinsic role of equity, healthcare workers risk doing major harm through the production of intervention-generated inequalities. Thus, any individual or system with an expectation of improving healthcare quality should be obligated by practice and policy to address healthcare equity.

If the community of healthcare providers, including physicians, who are often in positions of authority for the care of patients, does not leverage their organizational authority to create more equitable health outcomes for their patients, then we are dealing with serious issues. Change can take place at many levels: governmentally, organizationally, or nationally and even locally; but here we are discussing the change that we have greater influence over: the direct care of patients. Despite their position of power and authority in the healthcare system and with patients, our ongoing work tells us that many providers do not believe they have the power to effect the change for the equity they would like to see (this does not include those providers who have clearly stated they have no interest in equity, and it is “not their job” to address racism). Many providers also believe there is insufficient organizational support for equity work. Some providers even report being penalized or isolated for equity work or fear these consequences. Health care systems, on the other hand, believe that they offer significant authority and support to their providers to provide medical care that is fair and equal, yet it is unclear to them why inequities and disparities persist in their system, despite a lack of dedicated resources and strategies to improve equity.

The simplistic goal to treat people “fairly” is not enough. Fair is not always equitable, fairness only achieves equitable outcomes when the historical experiences of the patients we see has been fair, and they absolutely have not been—for many, their experiences have been systematically unfair and unjust, leading to the healthcare inequities we witness daily. To providers, we ask: have they forgotten and obfuscated the power that is held over the lives of patients by those who provide direct care? To healthcare systems, it is tantamount to realize that culture change only starts from leadership – letters and words of support are not sufficient; action and financial investment in change are the most important for staff and providers to believe in a new culture of healthcare equity. All these efforts ultimately benefit patients, which cascades to benefit the entire ecosystem of care – uplifting providers and building greater trust in the healthcare system. We must all appreciate that patients take risks to trust the healthcare system, including their providers. That trust is delicate and deserves the collective will of both providers and the healthcare system to hold and strengthen.

We acknowledge that the contradiction laid out above, between providers and healthcare systems, shows the impasse that stifles the great possibility of equitable care. There is at least another perspective, and that is the much broader financial system of healthcare that devalues equity and ultimately only uplifts the outsized value of relative-value units (RVUs). A devaluing of equity means it is not a priority and will not be achieved because the dollars do not make sense to do so. A fundamental question becomes, is there a financial health care approach that relies on equitable health outcomes as a metric for payment? There should be.

## Achieving healthcare equity

Considering the coalescing interests and perspectives laid out, it becomes clear that institutions, providers, and insurers struggle to center equitable care. So how do we achieve equity in our current conditions? The answer for us lies in the constant reminder of responsibilities that currently exist and some recommendations of what more we should do. Healthcare organizations and providers have the responsibility and authority to deliver equitable healthcare and can be held accountable in addressing healthcare inequities experienced by patients. Physicians play an important role in ensuring that their patients are provided with equitable healthcare, as a professional, ethical, and legal obligation. Not only are healthcare providers required to comply with federal non-discrimination policies when receiving federal funding (21), but there are growing calls within the medical community for equity to be included in the ethical and professional standards for physician training and care (22). Additionally, it would be beneficial to explore the Hippocratic oath with a much closer eye. From its original version, which has its flaws – it does read that physicians taking the Hippocratic oath must keep their patients safe “from harm and injustice” (23). There is room to also update the oath’s language to include health equity as an imperative principle in healthcare (22).

In an article from the AMA Journal of Ethics, Chen & Anderson elaborate on the gaps in professional obligations for transparency and accountability in health equity, explaining that “erosion of trust undermines patient-clinician relationships, exacerbates clinician burnout, contributes to moral injuries incurred by working in unjust systems, and diminishes health care quality and communities’ health” (22). They argue that, to improve health equity, “advocacy, leadership, and knowledge of health systems science and health policy are all key competencies that must be cultivated in clinicians to be prepared to meet their obligations to the public to eliminate inequity in health status and to promote access to health services”.

Chen & Anderson provide suggestions on how to improve clinician accountability to the public using equity-based standards, while also noting that physicians share responsibility with a cross-disciplinary team “to serve the public interest equitably” (22). Regarding the ethical obligations stated in the Hippocratic oath, they argue that the oath “should be regarded by those who take it as a professional obligation to draw upon the social status and cultural authority conferred by their profession to improve the material conditions (social determinants) of patients’ lives that undermine individual and community health status”.

While laws that apply to federally-funded healthcare systems “prohibit discrimination and require covered entities to provide individuals an equal opportunity to participate in a program activity, regardless of race, color, national origin, age, disability, religion, or sex (including pregnancy, sexual orientation, and gender identity)” (21), there are gaps in the enforcement of these laws and limitations in legal procedures to address health inequities. Legal cases involving discrimination in a healthcare setting are intentionally difficult to prove, despite many sources of data that reveal profound racial health inequities.

## Patients and families expect physicians to care about healthcare equity

Patients and families want and deserve equitable and quality healthcare. Any existing hospital-based equity policies are only realized when healthcare providers and staff connect policies with patients by facilitating the expression of those policies at the bedside. While lifesaving care is intended to be equitable because of consistently applied trauma care protocols, this is not often the patient experience. Patients experience inequities despite multiple opportunities to redress inequities during hospitalization and patients are aware. Too often, disregarding compassion, empathy, responsiveness, and respect for patient choice, needs, and values is legitimized and disincentivized as “idealistic” in deference to “practical” concerns related to efficiency, revenue generation, and meeting metrics; and this needs to change (24). We must all provide patient-centered care to ensure that patient values inform and guide medical decisions (15).

Mutual trust between healthcare workers and patients is also incredibly important as it improves communication, patient experiences, provider satisfaction, and wellbeing. Health equity and equitable care builds trust. Historical and current contexts of mistrust are often ignored during medical care which compromises patient and family safety. Incorporating cultural mediators and interpreter services as part of the care team may be helpful to increase provider-patient trust but does not obviate the need for provider responsibility to redress personal biases during medical care and advocate for equitable care in their health system. Initiatives might include active listening and responsiveness to patient concerns, improving health literacy pertaining to recognition, treatment and expectations of illness, and tailoring supports to optimize best outcomes (22). Trust develops when patients and families feel that their culture, values, and experiences are acknowledged and respected during medical care.

Equitable hiring practices in healthcare also matter (25). Achieving provider and staff diversity creates opportunities for trust-building during critical moments which leads to increased awareness of cultural considerations. This increased awareness allows providers and care teams who may share a similar identity to have more positive interactions with patients and families. Providers who endeavor to provide care that incorporates cultural humility acknowledge the value of equity in healthcare across cultures, and deliberately seek to understand patient needs, thereby increasing their “knowing” of patient experiences.

Receiving equitable care should be every patient’s right and achieving healthcare equity should be a societal and bedside imperative. This is a call to action for healthcare clinicians, institutions, and infrastructure to offer quality care grounded in equity. Bedside clinicians may not be fully equipped, potentially lacking knowledge, support, or bandwidth to actualize delivery of equitable healthcare. We call for alignment between patients, providers, and organizations to achieve this societal goal.

## Recommendations

While outside the scope of this work, further research is needed to conduct factor analyses that would advance a model for integrating equity into quality measures.

- Healthcare ecosystems should understand that our current medical economic model disincentivizes delivery of equitable healthcare and disempowers frontline clinicians in their efforts related to equity.
- Organizations should actively empower clinicians who wish to make change and to support them in their efforts, thereby building trust between providers and systems as well as between providers and patients.
- Healthcare ecosystems should actively empower patients to advocate for receipt of equitable healthcare delivery.
- Healthcare workers and organizations should adhere to stated institutional policies that espouse health equity to facilitate translation of stated goals into concrete outcomes.
- Healthcare systems need sustained and mandatory interactive training and education on providing equitable care and unraveling the inequities that currently exist in patient care.

## Example of success

One potential solution is organizational implementation of a health equity consult service, such as those found at the University of Michigan and Seattle Children’s Hospital that are being spearheaded at the University of Washington by author EL and the Office of Healthcare Equity. Such an effort would be preventive, collaborative, and build relationships between providers, patients and the medical institution. We call for the recognition of the need for additional efforts and interventions across healthcare systems and for their thoughtful and well-supported implementation.

## Data Availability

The original contributions presented in the study are included in the article/supplementary material, further inquiries can be directed to the corresponding author.
